# Levels of Circulating Fibroblast Growth Factor 23 (FGF23) and Prognosis in Cancer Patients with Bone Metastases

**DOI:** 10.3390/ijms20030695

**Published:** 2019-02-06

**Authors:** André Mansinho, Arlindo R. Ferreira, Sandra Casimiro, Irina Alho, Inês Vendrell, Ana Lúcia Costa, Rita Sousa, Catarina Abreu, Catarina Pulido, Daniela Macedo, Teresa R. Pacheco, Lurdes Correia, Luís Costa

**Affiliations:** 1 Medical Oncology Department, Centro Hospitalar Universitário Lisboa Norte, Hospital de Santa Maria, 1649-028 Lisbon, Portugal; andr3.m@gmail.com (A.M.); ajrsferreira@gmail.com (A.R.F.); inesvendrell@gmail.com (I.V.); c.ana.lucia4@gmail.com (A.L.C.), arita.sousa@gmail.com (R.S.); catarinaabreupm@hotmail.com (C.A.); danielavgmacedo@hotmail.com (D.M.); tr.pacheco@gmail.com (T.R.P.); 2 Luís Costa Lab, Instituto de Medicina Molecular, Faculdade de Medicina, Universidade de Lisboa, 1649-028 Lisbon, Portugal; scasimiro@medicina.ulisboa.pt (S.C.); irina.mpmad@gmail.com (I.A.); 3 Oncology Department, Hospital da Luz, 1500-650 Lisbon, Portugal; pulido.catarina@gmail.com; 4 Pathology Department, Centro Hospitalar Universitário Lisboa Norte, Hospital de Santa Maria, 1649-028 Lisbon, Portugal; lucorreia.mail@sapo.pt

**Keywords:** FGF23, bone metastases, cancer, prognosis

## Abstract

The fibroblast growth factor (FGF) signaling pathway plays a key role in tumorigenesis and is recognized as a potential therapeutic target. In this study, the authors aimed to assess the impact of serum FGF23 levels in the prognosis of patients with cancer and bone metastases from solid tumors. A cohort of 112 patients with cancer and metastatic bone disease were treated with bone-targeted agents (BTA). Serum baseline FGF23 was quantified by ELISA and dichotomized in FGF23^high^ and FGF23^low^ groups. Additionally, the association between FGF23 and overall survival (OS) and time to skeletal-related events (TTSRE) was investigated. Baseline characteristics were balanced between groups, except for the median urinary N-terminal telopeptide (uNTX) level. After a median follow-up of 26.0 months, a median OS of 34.4 and 12.2 months was found in the FGF23^low^ and FGF23^high^ groups, respectively (multivariate HR 0.18, 95% CI 0.07–0.44, *p* = 0.001; univariate HR 0.27, *p* = 0.001). Additionally, TTSRE was significantly longer for patients with FGF23^low^ (13.0 vs. 2.0 months, *p* = 0.04). Overall, this study found that patients with FGF23^low^ at baseline had longer OS and TTSRE. Further studies are warranted to define its role as a prognostic biomarker and in the use of drugs targeting the FGF axis.

## 1. Introduction

The fibroblast growth factor (FGF) protein family includes 22 members with diverse functions [1]. FGF23 is an approximately 32 kDa (251 amino acid) protein with the canonical N-terminal FGF homology domain and a novel 71 amino acid C-terminus [2], which acts as a phosphaturic factor and suppressor of 1α-hydroxylase activity in the kidney [3]. Although FGF23 is additionally present in other biologic structures, it is predominately expressed in osteocytes, which is the most abundant cells in bone [4]. Previous insights on FGF23 function have been mostly derived from mouse models, in which both administration of recombinant FGF23 and implantation of FGF23-overexpressing cells result in phosphaturia and hypophosphatemia [3]. Transgenic mice overexpressing FGF23 have decreased urinary phosphate reabsorption, hypophosphatemia, low serum 1,25-dihydroxyvitamin D [1,25(OH)2D] levels, and hyperparathyroidism [5,6].

FGF23 can bind to target cells via an FGF receptor (FGFR)—probably FGFR1—and its downstream signaling then requires a Klotho co-receptor [7]. Upon FGFR activation, type II sodium-phosphate cotransporters (NaPi-2a and NaPi-2c) in the kidney proximal tubule are down-regulated, leading to renal phosphate excretion [8]. Evidence suggests that the phosphaturic action of FGF23 is, to some extent, parathyroid hormone (PTH)-dependent. Subjects with hypoparathyroidism, typically with very low or undetectable PTH levels, have high serum phosphorus and FGF23, consistently with PTH requirements for a full phosphaturic FGF23 effect [9]. In addition to its action on NaPi-2a and NaPi-2c, FGF23 also regulates 1,25-vitamin D, by downregulating 1α-hydroxylase and up-regulating 24-hydroxylase, resulting in decreased 1,25-dihydroxy vitamin D [10].

Compelling evidence from human tumor sample analyses, in vitro studies, and animal models suggest that FGFRs are also relevant in cancer initiation and progression [10]. Due to its role in regulation of cell proliferation, migration, chemotaxis, morphogenesis, and angiogenesis, aberrant FGF signaling plays an important role in cancer development, tissue repair, and tumorigenesis. FGF23 is overexpressed in prostate cancer tissues and studies in prostate cancer cell lines have shown that it acts as an autocrine, paracrine, and/or endocrine growth factor [10]. Whereas exogenous FGF23 enhanced proliferation, invasion, and anchorage-independent growth in vitro, FGF23 knockdown decreased these phenotypes.

The role of FGF23 in a bone metastases-eliciting microenvironment remains unclear. Furthermore, the association between serum FGF23 and clinical outcomes in patients treated with bone-targeted agents (BTAs; i.e., bisphosphonates and denosumab) to prevent skeletal-related events (SREs) has not been described to date.

In the present study, the authors hypothesize that high levels of circulating FGF23 could negatively contribute to the prognosis of cancer patients with bone metastases, due to FGF23’s ability to promote cancer progression. Therefore, the primary objective of the study was to determine if FGF23 impacts the prognosis of cancer patients with bone metastases, by testing the association between FGF23 serum levels with overall survival (OS). The secondary objective was to investigate a possible association between serum FGF23 levels and time to skeletal-related events (TTSRE).

## 2. Results

### 2.1. Baseline Characteristics

Full data about patients’ characteristics at baseline was available for 103 patients. For the entire cohort, median (interquartile range [IQR]) FGF23 level was 32.09 (19.77–50.72) pg/mL and mean (standard deviation [SD]) FGF23 was 38.17 ± 26.15 pg/mL. When dichotomized into low and high categories, mean FGF23 was 26.15 pg/mL and 38.16 pg/mL, respectively. Overall, 19/112 (16.8%) patients were classified as FGF23^high^.

Baseline characteristics were balanced between groups (Table 1), except for median urinary N-terminal telopeptide (uNTX) levels, which were higher in the FGF23^high^ group (824.30 vs. 118.02 nmol bone collagen equivalents [BCE]/mmol creatinine, *p* = 0.040). Median time from beginning of BTA therapy was similar between groups (1.28 vs. 1.10 months, *p* = 0.161).

### 2.2. Survival Analysis

After a median (IQR) follow-up of 26.0 (13.0−47.0) months, 93 deaths (83.0%) were registered; 11 patients were lost to follow-up.

**Figure 1:**
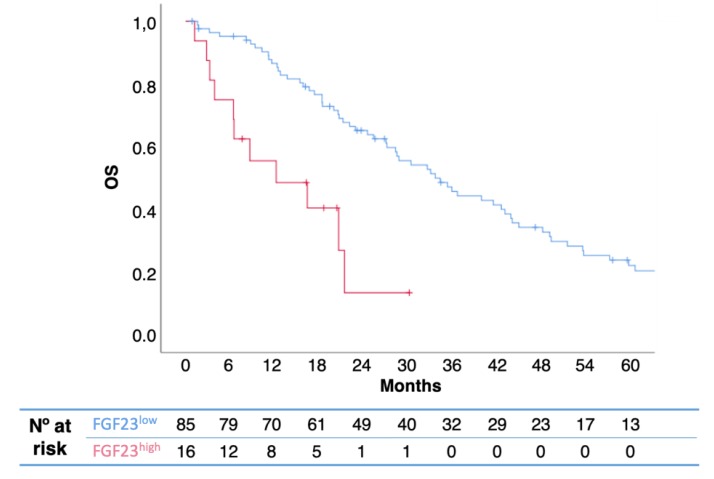
Kaplan-Meier overall survival (OS) curves, according to FGF23 levels. High levels of serum FGRF23 were associated with shorter patient survival. (*p* = 0.001; HR 0.27; 95% CI 0.14−0.55).

In univariate analysis, FGF23^high^ was associated with poorer OS than FGF23^low^ (median 12.2 vs. 34.4 months; *p* = 0.001; hazard ratio [HR] 0.27; 95% confidence interval [CI] 0.14−0.55) (Figure 1). In multivariate analysis including pre-therapeutic prognostic factors (extra-bone involvement, uNTX, presence of bone fractures, and serum levels of calcium), FGF23^high^ remained predictive of OS (Table 2). When tumor types were evaluated separately, no statistically significant correlation with prognosis was observed, what can be due to the small sample size of subgroup analyses.

**Figure 2:**
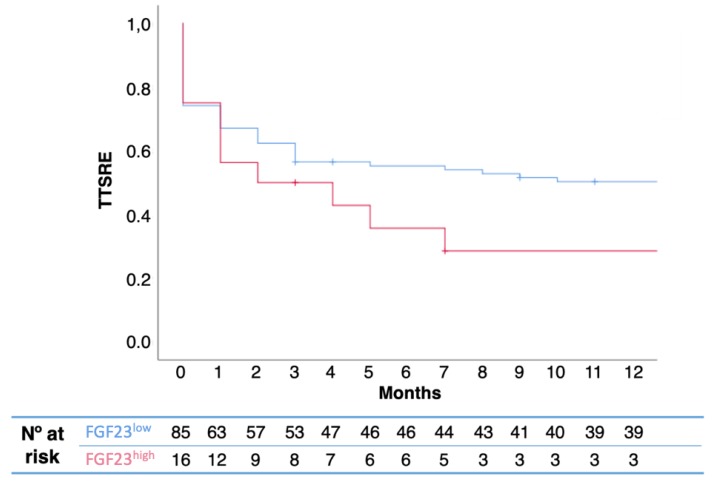
Kaplan-Meier TTSRE curves, according to FGF23 levels. High levels of serum FGRF23 were associated with shorter time to SREs (13.0 vs. 2.0 months, *p* = 0.04). P-value was calculated using log-rank test.

In the Kaplan-Meier model, elevated FGF23 was also predictive of a shorter TTSRE (13.0 vs. 2.0 months, *p* = 0.04) (Figure 2). The proportion of SRE was not statistically different between groups (Table 3).

## 3. Discussion

Most biologic prognostic factors derived from bone microenvironment identified in patients with bone metastases are not tumor-type dependent. Indeed, biomarkers as uNTX and alkaline phosphatase (ALP), reflecting both osteoclast and osteoblast activity, are strong predictors of survival in patients with bone metastases across several different tumor types [11].

Interestingly, FGFR signaling has a relevant role in advanced cancer stages, particularly when cancer cells survive and become resistant to hormonal agents, as in androgen deprivation-resistant prostate [12] and endocrine therapy-resistant breast [13] cancers.

Due to the relevance of FGFR signaling when considering endocrine resistance in the most frequent bone metastases-eliciting tumors (as prostate and breast) and the role of FGF23 in bone metabolism, authors of the present work considered that it was relevant to study the correlation of serum FGF23 with survival and TTSREs in patients with bone metastases.

The role of FGF23 in the pathogenesis of tumor-induced osteomalacia (TIO)—also known as oncogenic osteomalacia—is well described [14]. TIO is characterized by decreased renal tubular reabsorption of phosphate, low levels of active vitamin D, and chronic hypophosphatemia, ultimately leading to improper bone mineralization.

In this exploratory cohort, patients in the FGF23^high^ group had a shorter OS and TTSRE, which represents the first evidence of the prognostic relevance of serum FGF23 in patients with bone metastases, as far as authors are aware.

This is particularly important as FGFR signaling is currently under investigation as a potential target in cancer. In fact, FGFR antagonists have shown activity in prostate cancer [15] and have been approved to treat clear cell carcinoma and thyroid medullar carcinoma. In breast cancer, results have been less encouraging, possibly due to the lack of biomarkers to select patients that may benefit from FGFR inhibitors [16].

This study has some limitations: (i) it is a small retrospective analysis of a prospective cohort, thus subject to residual confounding; (ii) it included different tumor types (being breast cancer the most predominant); and (iii) expression of FGF23 in metastatic tissue was not assessed. Although not statistically significant, there was a numeric imbalance between the two groups regarding tumor types and presence of extraskeletal metastases. However, its findings suggest that the investigation on the role of FGF23 as a potential prognostic biomarker in patients with bone metastases should be pursued and deepened.

## 4. Materials and Methods

### 4.1. Study Population

In this prospective cohort study, 112 consecutive patients were diagnosed and treated at the Medical Oncology Department of Hospital de Santa Maria. Patients had metastatic bone disease from solid tumors and were treated with BTAs (either denosumab or zoledronate). They were consecutively enrolled upon baseline metastatic diagnosis confirmation and prospectively evaluated for disease progression and incidence of SREs every three to four months for one year. Urine and whole blood samples were collected at baseline and during treatment. At the time of diagnosis, overall cancer treatment was provided as per institutional guidelines and in accordance with international oncology society recommendations. Clinical information was retrospectively collected, including tumor type, concomitant therapy, number and timing of SREs, calcium level, date of disease progression (bone and extra-bone progression), and survival. SREs were identified as pathologic fracture, need for radiotherapy to treat bone metastases, spinal cord compression, or surgery to bone. Ethical approval for this study was granted by institutional review boards on 19 December 2013 and compliance with national regulations was observed.

### 4.2. uNTX and FGF23 Determination

uNTX was quantified using Osteomark NTx Urine enzyme-linked immunosorbent assay (Alere, Waltham, MA) according to the manufacturer’s instructions. Urinary levels of NTX in BCEs were expressed as urine creatinine excretion ratio. FGF23 was quantified by ELISA (Kainos Laboratories Inc., Tokyo, Japan), according to the manufacturer’s instructions. 

### 4.3. Statistical Analysis

FGF23 was dichotomized in two levels (FGF23^high^ and FGF23^low^) using a sample mean plus one standard deviation as cut-off. Descriptive statistics of baseline clinical and pathologic characteristics according to FGF23 levels were performed. Given the exploratory nature of the study, no specific sample size was predefined. 

Median and IQR was reported for continuous variables and absolute and relative frequencies, for categorical variables. To compare categorical variables among groups, χ^2^ and Fisher exact tests were used when appropriate. The Mann-Whitney test was used to compare medians.

Outcome measures included OS and TTSRE. OS was defined as time between BTA start and death, irrespective of the cause. TTSRE was defined as time between BTA start and first SRE. Patients without a vital status change by January 2018 were right censored.

Median OS and TTSRE were obtained using Kaplan-Meier methods and log-rank test was used to compare group outcomes. Cox regression model was used to perform univariate and multivariate assessment of the effect of investigated parameters in duration of response and prognosis.

All statistical tests were two-tailed and statistical significance was assumed when *p* values were inferior to 0.05. IBM’s SPSS statistics v.25 was used for the statistical analysis.

## 5. Conclusions

In this exploratory cohort, patients in the FGF23^high^ group had a shorter OS and TTSRE compared with those on the FGF23^low^ group. Further studies are warranted to define FGF23 role as a prognostic biomarker and potential predictor of response to drugs targeting the FGF23-FGFR axis. This study’s results suggest that new therapeutic agents targeting FGF23 transcription and/or degradation to either increase or lower circulating FGF23 levels, as well as recombinant FGF23- or FGF23-blocking antibodies, could be explored regarding their eventual role in the treatment of bone metastatic disease.

## Figures and Tables

**Table 1:** Baseline characteristics of the cohort included.

Characteristic	FGF23^low^ (*n* = 94)	FGF23^high^ (*n* = 18)	*p*
**Age**, years			
Average ± SD	57.4 ± 14.1	57.9 ± 5.6	0.360
Median (IQR)	57.5 (45.9–68.3)	58.9 (56.2–61.9)	
**Tumor type**, *n* (%)			
Breast	58 (65.2)	8 (53.3)	0.096
Prostate	16 (18.0)	1 (6.7)	
Others (Renal Cell Carcinoma—9; Biliary adenocarcinoma—1; Gastric adenocarcinoma—1; Sarcoma—3; Urothelial—4; Lung adenocarcinoma—1; Neuroendocrine—1; Cervix squamous carcinoma—1)	15 (16.9)	6 (40.0)	
**Extraskeletal metastases**, *n* (%)			
Yes	51 (60.0)	12 (80.0)	0.161
No	34 (40.0)	3 (20.0)	
**Time to BTA**, median months	1.10	1.28	0.161
**uNTX**, nmol BCE/mmol creatinine			
Median (IQR)	118.0	824.3	0.040
NTX^high^, n (%)	45 (52.9)	6 (66.7)	0.501
NTX^low^, n (%)	40 (47.1)	3 (33.3)	
**Calcium**, *n* (%)			
Hypercalcemia	15 (17.0)	6 (37.5)	0.113
Normal range	66 (75.0)	10 (62.5)	
Hypocalcemia	7 (8.0)	0 (0.0)	
Median (IQR)	9.7 (9.4–10.1)	10.5 (9.9–11.05)	0.883

SD: standard deviation; IQR: interquartile range; BTA: bone-targeted agents; uNTX: urinary N-terminal telopeptide; BCE: bone collagen equivalents; FGF: fibroblast growth factor.

**Table 2:** Cox regression models for OS.

	Median (95% CI)	Univariate HR (95% CI)	*p*	Multivariate HR (95% CI)	*p*
**FGF23**					
FGF23^low^	34.4 (26.4–42.3)	0.27 (0.14–0.55)	0.001	0.18 (0.07–0.44)	0.001
FGF23^high^	12.2 (0.0–25.1)				
**Hypercalcemia**					
No	34.4 (22.8–45.9)	0.55 (0.32–0.97)	0.036	0.62 (0.34–1.15)	0.133
Yes	18.5 (6.9–30.0)				
**Pathologic fractures**					
No	28.8 (19.3–38.3)	1.03 (0.60–1.77)	0.911	1.13 (0.63–2.00)	0.689
Yes	34.4 (13.8–55.0)				
**Extraskeletal metastases**					
No	43.9 (35.6–52.2)	0.63 (0.39–1.02)	0.058	0.62 (0.37–1.04)	0.070
Yes	22.9 (17.5–28.3)				

**Table 3:** SRE occurrence by FGF group.

SRE, *n* (%)	FGF_low_	FGF_high_	*p* Value
Yes	54 (57.4)	6 (33.3)	0.603
No	40 (42.6)	12 (66.7)	
Median (IQR)	1 (0.0–2.0)	1 (1.0–1.5)	0.419

SRE: skeletal-related events.

## Abbreviations

ALP	alkaline phosphatase
BCE	bone collagen equivalent
BTA	bone-targeted agents
ELISA	enzyme-linked immunosorbent assay
FGF	fibroblast growth factor
FGF	fibroblast growth factor receptor
HR	hazard ratio
IQR	interquartile range
OS	overall survival
PTH	parathyroid hormone
SRE	skeletal-related event
TIO	tumor-induced osteomalacia
TTSRE	time to skeletal-related events
uNTX	urinary N-terminal telopeptide
